# Unraveling Aβ-Mediated Multi-Pathway Calcium Dynamics in Astrocytes: Implications for Alzheimer’s Disease Treatment From Simulations

**DOI:** 10.3389/fphys.2021.767892

**Published:** 2021-10-28

**Authors:** Langzhou Liu, Huayi Gao, Alexey Zaikin, Shangbin Chen

**Affiliations:** ^1^Britton Chance Center for Biomedical Photonics, Wuhan National Laboratory for Optoelectronics, Huazhong University of Science and Technology, Wuhan, China; ^2^MoE Key Laboratory for Biomedical Photonics, School of Engineering Sciences, Huazhong University of Science and Technology, Wuhan, China; ^3^Institute of Information Technologies, Mathematics and Mechanics, Lobachevsky State University of Nizhny Novgorod, Nizhny Novgorod, Russia; ^4^Institute for Women’s Health and Department of Mathematics, University College London, London, United Kingdom; ^5^World-Class Research Center “Digital Biodesign and Personalized Healthcare”, Sechenov First Moscow State Medical University, Moscow, Russia

**Keywords:** Aβ, Alzheimer’s disease, astrocyte, calcium oscillations, dyshomeostasis, therapy

## Abstract

The accumulation of amyloid β peptide (Aβ) in the brain is hypothesized to be the major factor driving Alzheimer’s disease (AD) pathogenesis. Mounting evidence suggests that astrocytes are the primary target of Aβ neurotoxicity. Aβ is known to interfere with multiple calcium fluxes, thus disrupting the calcium homeostasis regulation of astrocytes, which are likely to produce calcium oscillations. Ca^2+^ dyshomeostasis has been observed to precede the appearance of clinical symptoms of AD; however, it is experimentally very difficult to investigate the interactions of many mechanisms. Given that Ca^2+^ disruption is ubiquitously involved in AD progression, it is likely that focusing on Ca^2+^ dysregulation may serve as a potential therapeutic approach to preventing or treating AD, while current hypotheses concerning AD have so far failed to yield curable therapies. For this purpose, we derive and investigate a concise mathematical model for Aβ-mediated multi-pathway astrocytic intracellular Ca^2+^ dynamics. This model accounts for how Aβ affects various fluxes contributions through voltage-gated calcium channels, Aβ-formed channels and ryanodine receptors. Bifurcation analysis of Aβ level, which reflected the corresponding progression of the disease, revealed that Aβ significantly induced the increasing [Ca^2+^]_*i*_ and frequency of calcium oscillations. The influence of inositol 1,4,5-trisphosphate production (IP_3_) is also investigated in the presence of Aβ as well as the impact of changes in resting membrane potential. In turn, the Ca^2+^ flux can be considerably changed by exerting specific interventions, such as ion channel blockers or receptor antagonists. By doing so, a “combination therapy” targeting multiple pathways simultaneously has finally been demonstrated to be more effective. This study helps to better understand the effect of Aβ, and our findings provide new insight into the treatment of AD.

## Introduction

Alzheimer’s disease (AD) is currently the most common neurodegenerative disease and is the major dementia type, which accounts for 60–70% of cases (Prince, 2015; Canter et al., 2016). The Alzheimer Disease International estimated in 2019 that over 50 million people were living with dementia and this number is expected to exceed 150 million by 2050 (Lynch, 2020). The disease has already caused incalculable losses worldwide. Little is known about the complex pathophysiology of AD, and thus, there is no cure. Pathological characteristics of accumulation of amyloid β-peptide (Aβ), is considered to be drivers in AD pathogenesis (Hardy and Selkoe, 2002; Birch, 2014).

Astrocytes were historically considered to provide support for neurons (Carmignoto and Gómez-Gonzalo, 2010). Since they were found to be involved in many brain functions and neurodegenerative diseases such as AD and Parkinson’s disease etc., astrocytes have become a hot topic in neuroscience research over the past few decades (Tewari and Majumdar, 2012; Tewari and Parpura, 2013; Bazargani and Attwell, 2016). In cultures of mixed neurons and astrocytes treated with Aβ, astrocytes always exhibit pathological alterations before neuronal death suggesting that astrocytes appear to be the primary target of Aβ (Abramov et al., 2003). Their role as protector and housekeeper in central nervous system is universally acknowledged, however, Aβ impairs important supportive astrocyte functions in AD cases (Verkhratsky and Nedergaard, 2018).

Astrocytes do not generate electrical signals like neurons (Wu et al., 2015). However, the concept of “cellular excitability” in astrocytes has been recently formalized to describe the changes in cytosolic Ca^2+^ concentration in response to chemical or mechanical stimulation (Verkhratsky, 2019). For example, they encode synaptic information via the modulation of intracellular calcium dynamics in response to synaptic activity (De Pittà et al., 2009). However, this kind of Ca^2+^ homeostasis can be disrupted by Aβ, whether in neurons or astrocytes, especially its soluble oligomeric form is more harmful (Demuro et al., 2010). In an AD mouse model, astrocytes displayed higher basal astrocyte Ca^2+^ levels and increased transient Ca^2+^ signals (Kuchibhotla et al., 2009).

Aβ is known to interfere with multiple calcium fluxes in astrocytes. Aβ oligomers deposition not only form pores in the lipid bilayer permeable to cationic ions, but also directly or indirectly activate L-type Ca_*V*_, which increases the concentration of intracellular Ca^2+^ (Alves et al., 2019). Additionally, the expression of astroglial mGluR_5_ is up-regulated by exposure to Aβ (Lim et al., 2013). Inside astrocytes, Aβ can induce endoplasmic reticulum (ER) Ca^2+^ release through ryanodine receptors (RyRs) and inositol triphosphate receptors (IP_3_Rs) (Alberdi et al., 2013). How to combine these different findings to understand the big picture is crucial for further research (Markowetz, 2017). However, it is experimentally very difficult to investigate the interactions of many mechanisms of Ca^2+^ dyshomeostasis (Cutsuridis and Moustafa, 2017), e.g., the limitations of Ca^2+^ indicators and imaging techniques.

Simulation based on the mathematical model has been an invaluable tool to investigate complex interactions. There are hundreds of computational models on astrocyte Ca^2+^ dynamics and homeostasis either in a single astrocyte or in astrocyte networks or in neuron-astrocyte synapses (Manninen et al., 2018). Here, we focus primarily on modeling efforts in single astrocyte. Most of them studied Ca^2+^ oscillations, while a small part of them modeled spontaneous Ca^2+^ activity (Lavrentovich and Hemkin, 2008; Riera et al., 2011b); some assessed neurotransmitter-evoked Ca^2+^ excitability (De Pittà et al., 2009; Dupont et al., 2011). These computational studies are mostly based on classic models, including components for calcium-induced calcium release (CICR) and the sarco-endoplasmic Ca^2+^ ATPase pump (SERCA). In astrocytes, intracellular Ca^2+^ oscillations depend mainly on CICR, while Ca^2+^ influx from extracellular space via receptors or channels on membrane such as voltage-gated calcium channels (VGCCs) has also been linked with Ca^2+^ oscillations (Lavrentovich and Hemkin, 2008; Zeng et al., 2009). Taheri et al. (2017) modeled capacitive Ca^2+^ entry, which is mediated via store-operated Ca^2+^ channels. Ding et al. (2018) constructed two stochastic models, one describing the VGCC channel noise and the other describing the stochastic IP_3_R dynamics. Recently, some modeling work has addressed the effect of Aβ on Ca^2+^ dynamics in generic cells (Latulippe et al., 2018). We established an Aβ-mediated calcium signaling model in astrocytes for the pilot study (Gao et al., 2020). All the models have useful implications for understanding Ca^2+^ signaling. However, no model has integrated the abovementioned putative mechanisms (Abramov et al., 2003; Alberdi et al., 2013; Lim et al., 2013; Alves et al., 2019) to simulate Ca^2+^ dynamics in astrocytes. The synergistic effect of the different Ca^2+^ fluxes on Ca^2+^ dynamics is not well understood so far. In particular, the current development of the theme lacks results regarding restoring the Ca^2+^ homeostasis in astrocytes during the progression of AD.

To date, the treatment of AD has remained a challenge. Although some promising drugs are under continuous development, clinical trials in recent years fail to get satisfied results (Qian et al., 2015). The currently approved anti-AD drugs fall into two types: cholinesterase inhibitors (donepezil, rivastigmine and galantamine) and N-methyl-D-aspartic acid receptor antagonists (memantine) (Aupperle, 2006). On the one hand, these drugs can only relieve symptoms but do not prevent the progression of AD, suggesting that their targets might not be the disease origin (Liu et al., 2019). On the other hand, memantine can prevent NMDAR-mediated Ca^2+^ flux indicating that other Ca^2+^ mechanisms may be a potential target for the treatment of AD when they have been demonstrated to play a proximal role in AD.

Here, a comprehensive model integrating multiple Aβ-affected Ca^2+^ pathways is proposed. This model of astrocytes describes Ca^2+^ signals as individual Ca^2+^ transport pathways rather than a macroscopic flow of Ca^2+^, including both intracellular release and extracellular influx. With this computational model, we can begin to study how Aβ affects each source of Ca^2+^ through various pathways. Recent wet-lab data are also incorporated into modeling work to obtain insights into AD treatment (Sadick and Liddelow, 2019). The explicit intention is to test the specific treatment strategy for AD.

## Materials and Methods

### Biophysical Model

**FIGURE 1:**
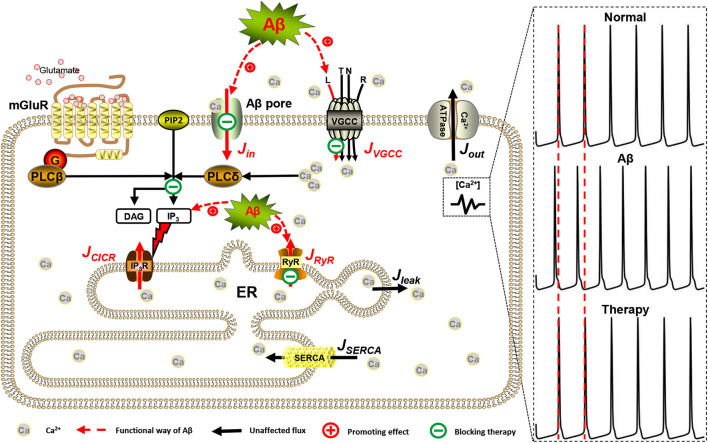
Mechanisms involved in intracellular Ca^2+^ dysregulation in AD. In this model, *J*_*VGCC*_ and *J*_*in*_ are Ca^2+^ influxes from the extracellular space. The two separate mechanisms involved in the process of Ca^2+^ release from the ER via IP_3_Rs and RyRs are *J*_*CICR*_ and *J*_*RyR*_, respectively. *J*_*SERCA*_ represents the SERCA pump of the ER refilling the ER by pumping Ca^2+^ back from the cytosol. *J*_*leak*_ is the leakage Ca^2+^ flux from the ER into the cytosol. *J*_*out*_ is Ca^2+^ efflux by Ca^2+^-ATPase pump. Red lines represent the functional pathway of Aβ, and green lines represent the inhibitory effects of drugs.

Intracellular Ca^2+^ levels are modulated by influx from the extracellular space or controlled release from intracellular Ca^2+^ stores such as the ER. Generally, Ca^2+^ entry into astrocytes includes active transport by different types of VGCCs distributed in the membrane and passive leakage. In astrocytes, however, IP_3_-dependent CICR from the ER is considered the primary mechanism responsible for intracellular Ca^2+^ dynamics (Agulhon et al., 2008). CICR is essentially controlled by efflux from the ER to the cytoplasm that is mediated both by IP_3_R and RyR and influx into the ER, which is due to the action of SERCA pumps. Aβ can interfere with some of these Ca^2+^ fluxes and the detailed modeling methods for Aβ are described in section “Aβ Assumption.” So, the whole-cell model is shown in Figure 1.

#### Intracellular Ca^2+^ Dynamics

We describe the model by tracking the flux in and out of the cytoplasm. Then, the change in intracellular Ca^2+^ is governed by


(1) $$d\,{\left[C\,a^{2+}\right]}_{i}/d\,t=J_{V\,G\,C\,C}-J_{o\,u\,t}+J_{i\,n}+J_{C\,I\,C\,R}+J_{R\,y\,R}+J_{l\,e\,a\,k}-J_{S\,E\,R\,C\,A},$$

(2) $$d\,{\left[C\,a^{2+}\right]}_{E\,R}/d\,t=\left(J_{S\,E\,R\,C\,A}-J_{C\,I\,C\,R}-J_{R\,y\,R}-J_{l\,e\,a\,k}\right)/c_{1},$$

where [*Ca*^2 +^]_*i*_ and [Ca^2 +^]_*ER*_denote the concentration of Ca^2+^ in the cytoplasm and ER, respectively. c_1_is the ratio of ER volume to the cytoplasmic volume. We assumed a spatially homogeneous astrocyte whose volume was fixed. As such, the ER and cytoplasm are simplified as two points of the cell for better quantifying and identifying key mechanisms behind certain Ca^2+^ fluxes.

The term *J*_*VGCC*_ represents the pathway of Ca^2+^ influx through four types of VGCCs, including L-, N-, T-, and R-types. When the volume of the cell is constant, the Ca^2+^ flux and current are related by the equations (Zeng et al., 2009):


(3) $$J_{V\,G\,C\,C}=-\frac{I_{V\,G\,C\,C}}{z\,F\,V_{a\,s\,t}},$$

(4) $$I_{V\,G\,C\,C}=I_{C\,a,L}+I_{C\,a,T}+I_{C\,a,N}+I_{C\,a,R},$$

where *I*_*VGCC*_ is the VGCC-conducted Ca^2+^ current, and *I*_*Ca,L*_, *I*_*Ca,T*_, *I*_*Ca,N*_, *I*_*Ca,R*_ represent the current through different types of channels. *z* is the valence of Ca^2+^, *F* is the Faraday constant and *V*_*ast*_ is the volume of the astrocyte. The concrete formula for every type of calcium current is given in detail in Table 1.

**TABLE 1:** Details of the voltage-gated calcium channels.

Channel type	Equation of channel dynamics
T-type	*I*_*Ca*,*T*_=*g*_*T*_*m*_*T*_(*h*_*Tf*_ + 0.04*h*_*Ts*_)(*V*_*m*_−*E*_*Ca*_)
	${\bar{m}}_{T}=\frac{1}{1+e^{-\left(V_{m}+63.5\right)/1.5}}\,{\bar{h}}_{T}=\frac{1}{1+e^{\left(V_{m}+76.2\right)/3}}$
L-type	*I*_*Ca*,*L*_=*g*_*L*_*m*_*L*_*h*_*L*_(*V*_*m*_−*E*_*Ca*_)
	${\bar{m}}_{L}=\frac{1}{1+e^{-\left(V_{m}+50\right)/3}}\,{\bar{h}}_{L}=\frac{0.00045}{0.00045+{\left[C\,a^{2+}\right]}_{i}}$
N-type	*I*_*Ca*,*N*_=*g*_*N*_*m*_*N*_*h*_*N*_(*V*_*m*_−*E*_*Ca*_)
	${\bar{m}}_{N}=\frac{1}{1+e^{-\left(V_{m}+45\right)/7}}\,h_{N}=\frac{0.0001}{0.0001+{\left[C\,a^{2+}\right]}_{i}}$
R-type	*I*_*Ca*,*R*_=*g*_*R*_*m*_*R*_*h*_*R*_(*V*_*m*_−*E*_*Ca*_)
	${\bar{m}}_{R}=\frac{1}{1+e^{-\left(V_{m}+10\right)/10}}\,{\bar{h}}_{R}=\frac{1}{1+e^{\left(V_{m}+48\right)/5}}$

**Here*,*g* *is the conductance of the channel*,*V*_*m*_ *is the resting membrane potential*,*E*_*Ca*_ *is the Nernst potential of the VGCC and is expressed as* $E_{C\,a}=\frac{R\,T}{z\,F}\,l\,n\,\frac{{\left[C\,a^{2+}\right]}_{o}}{{\left[C\,a^{2+}\right]}_{i}}$, *m and* *h* *are gating variables;* $\bar{m}$, $\bar{h}$ *are steady states of the channel activation and inactivation variables, respectively.**

*J*_*in*_ is a passive leakage from the extracellular space. Riera et al. (2011a) used a heuristic strategy to determine *J*_*in*_ = 0.036 μM/s for healthy astrocytes:


(5) $$J_{i\,n}=v_{5}.$$

To represent the kinetics of the IP_3_R, we used a simplification form (Ullah et al., 2006) described as


(6) $$J_{C\,I\,C\,R}=v_{1}\,m^{3}\,n^{3}\,h^{3}\,\left({\left[C\,a^{2+}\right]}_{E\,R}-{\left[C\,a^{2+}\right]}_{i}\right),$$

where *v_1_* determines the maximal rate of transported Ca^2+^, *m*, *n* and *h* are gating variables of IP_3_R. The first two are assumed to have instantaneous kinetics, *m*=*m*_∞_, *n*=*n*_∞_, while *h* obeys Hodgkin-Huxley formalism. They are specified as follows:


(7) $$m_{\infty }=\frac{\left[I\,P_{3}\right]}{\left[I\,P_{3}\right]+d_{1}},$$

(8) $$n_{\infty }=\frac{{\left[C\,a^{2+}\right]}_{i}}{{\left[C\,a^{2+}\right]}_{i}+d_{5}},$$

(9) $$d\,h/d\,t=a_{2}\,d_{2}\,\frac{\left[I\,P_{3}\right]+d_{1}}{\left[I\,P_{3}\right]+d_{3}}\,\left(1-h\right)-a_{2}\,{\left[C\,a^{2+}\right]}_{i}\,h,$$

*d_1_* and *d_5_* determine the dissociation of IP_3_ and Ca^2+^ by the channel’s subunits, whereas *d_2_* and *d_3_* is the inactivation dissociation constant of Ca^2+^ and IP_3_, respectively. *a_2_* determines the IP_3_R binding rate for Ca^2+^ inhibition.

The SERCA pump rate can be taken as an instantaneous function of [Ca^2+^]_*i*_ by using a Hill-type kinetic model (Ullah et al., 2006):


(10) $$J_{S\,E\,R\,C\,A}=v_{3}\,\frac{{\left[C\,a^{2+}\right]}_{i}^{2}}{{\left[C\,a^{2+}\right]}_{i}^{2}+k_{3}^{2}},$$

where *v_3_* represents the maximum SERCA pump flux and *k_3_* is the dissociation constant of Ca^2+^ to SERCA.

To model RyR, we modified the previous model (Friel, 1995) by increasing the effect of [Ca^2+^]_*i*_ and took the following form:


(11) $$J_{R\,y\,R}=\left(k_{0}+\frac{k_{2}\,{\left[C\,a^{2+}\right]}_{i}^{3}}{k_{d}^{3}+{\left[C\,a^{2+}\right]}_{i}^{3}}\right)\,\left({\left[C\,a^{2+}\right]}_{E\,R}-{\left[C\,a^{2+}\right]}_{i}\right),$$

where *k_0_* is the zero calcium concentration level leak. This parameter is usually used to ensure a physiologically meaningful resting Ca^2+^ level (Dupont et al., 2016). Furthermore, *k_2_* is the maximal rate of the channel, *k_d_* corresponds to the RyR channel sensitivity for the CICR.

The leakage flux from the ER is assumed to be proportional to the Ca^2+^ gradient across the ER membrane by *v_2_*, the maximal rate of Ca^2+^ leakage (Ullah et al., 2006).


(12) $$J_{l\,e\,a\,k}=v_{2}\,\left({\left[C\,a^{2+}\right]}_{E\,R}-{\left[C\,a^{2+}\right]}_{i}\right).$$

Finally, we considered Ca^2+^ extrusion flux as described (Ullah et al., 2006):


(13) $$J_{o\,u\,t}=k_{1}\,{\left[C\,a^{2+}\right]}_{i},$$

where *k_1_* is the rate constant of calcium extrusion.

#### Generation/Degradation of Cytosolic IP_3_

IP_3_ is the second messenger involved in G protein-coupled receptor-mediated signal transduction. In astrocytes, IP_3_ is produced by hydrolysis of phosphatidylinositol 4,5-bisphosphate by two phosphoinositide-specific phospholipase C (PLC) isoenzymes, PLCβ and PLCδ (Rebecchi and Pentyala, 2000). Therefore, the IP_3_ dynamic is described as:


(14) $$d\,\left[I\,P_{3}\right]/d\,t=J_{P\,L\,C\,\beta }+J_{P\,L\,C\,\delta }-k_{d\,e\,g}\,\left[I\,P_{3}\right],$$

where *J*_*PLCβ*_ and *J*_*PLCδ*_ are PLCβ- and PLCδ-dependent IP_3_ production, respectively. *k*_*deg*_ represents the rate of IP_3_ degradation.

PLCβ is primarily controlled by external glutamate stimulation (De Pittà et al., 2009). So *J*_*PLCβ*_ can be modeled as follows (De Pittà et al., 2009):


(15) $$J_{P\,L\,C\,\beta }=v_{\beta }\,\frac{g^{0.7}}{g^{0.7}+{\left(k_{R}+k_{P}\,\frac{{\left[C\,a^{2+}\right]}_{i}}{{\left[C\,a^{2+}\right]}_{i}+k_{\pi }}\right)}^{0.7}},$$

where *v*_β_ is the maximal PLCβ rate, *g* is the concentration of glutamate, we set this value to be *g* = 1 μM. *k_R_* is glutamate affinity and *k_P_* is the Ca^2+^/PLC-dependent inhibition factor and *k*_π_ controls Ca^2+^ affinity of PLC.

In contrast, PLCδ is essentially activated by increased intracellular Ca^2+^ levels (Rhee and Bae, 1997) and is written as (De Young and Keizer, 1992):


(16) $$J_{P\,L\,C\,\delta }=v_{4}\,\frac{{\left[C\,a^{2+}\right]}_{i}+\left(1-\alpha \right)\,k_{4}}{{\left[C\,a^{2+}\right]}_{i}+k_{4}},$$

where *v_4_* is the maximum rate of IP_3_ production, and *k_4_* is the dissociation constant for Ca^2+^ stimulation of IP_3_ production. Here α is used to investigate the relative effect of Ca^2+^ stimulation of PLCδ on IP_3_ production. For example, if α = 0, the IP_3_ production rate is *v_4_*, which is independent of [Ca^2+^]_*i*_.

### Aβ Assumption

Exposure of astrocytes to Aβ was reported to trigger [Ca^2+^]_*i*_ transients and [Ca^2+^]_*i*_ oscillations (Alberdi et al., 2013). Such effects may involve various Ca^2+^ entry pathways as well as Ca^2+^ release from ER (Abramov et al., 2003; Alberdi et al., 2013). These experimental findings have provided useful insights for us to make our Aβ assumption. However, in the real condition, the accumulation of Aβ can occur over months, years, and even decades which does not match the short timescale of changes in Ca^2+^. To solve this issue, we assumed a fixed level of Aβ concentration in our model using the parameter *a* which corresponds to the certain stage of the progression of AD. For example, a small value of *a* may reflect a low level of Aβ representing the early stage of the disease. By changing *a*, we can easily investigate the effect of Aβ on different progressions of AD.

A study has demonstrated that L-type channels might be activated by Aβ, and increased expression of L-type channels is associated with Aβ-positive plaques (Alves et al., 2019). To do this, we altered the original form of *I*_*Ca,L*_:


(17) $$I_{C\,a,L}=\left(g_{L}+k_{V\,G\,C\,C}\,a\right)\,m_{L}\,h_{L}\,\left(V_{m}-E_{C\,a}\right),$$

where *k*_*VGCC*_ controls the strength of the effect of Aβ on the channels.

Besides, Aβ-formed channels on the plasma membrane can also trigger additional Ca^2+^ influx into the cytoplasm (Demuro et al., 2010). In order to incorporate the possible influence, we included another term in*J*_*in*_:


(18) $$J_{i\,n}=v_{5}+k_{i\,n}\,a^{k},$$

where *k*_*in*_ represents a rate constant (De Caluwé and Dupont, 2013), or, in other words, the strength of Aβ in this study, and *k* is the cooperativity coefficient.

Although some studies have elucidated the role of RyRs in regulating Ca^2+^ disruption in AD (Stutzmann et al., 2006; Goussakov et al., 2010; Briggs et al., 2013), data on the contributions of Ca^2+^ flux through the RyR in the presence of Aβ are minimal. Given that Aβ can increase the channel open probability, we decided to revise the expression as follows:


(19) $$J_{R\,y\,R}=\left(k_{0}+\frac{k_{2}\,{\left[C\,a^{2+}\right]}_{i}^{3}}{{\left(k_{d}+k_{R\,y\,R}\,a\right)}^{3}+{\left[C\,a^{2+}\right]}_{i}^{3}}\right)\,\left({\left[C\,a^{2+}\right]}_{E\,R}-{\left[C\,a^{2+}\right]}_{i}\right),$$

where *k*_*RyR*_ represents the strength of Aβ.

In addition to the direct effect on Ca^2+^ fluxes, Aβ can also alter IP_3_ levels. On the one hand, Aβ up-regulates the expression of astroglial mGluR_5_, therefore, affects downstream IP_3_ production (Renner et al., 2010). On the other hand, intracellular Aβ oligomers induce Ca^2+^ liberation from the ER via IP_3_Rs by stimulating PLC-mediated IP_3_ production (Demuro and Parker, 2013). Based on these findings, we adapted equations (15) and (16), assuming that the glutamate- and Ca^2+^-dependent IP_3_ production would take the following forms:


(20) $$J_{P\,L\,C\,\beta }=\left(v_{\beta }+k_{P\,L\,C\,\beta }\,a\right)\,\frac{g^{0.7}}{g^{0.7}+{\left(k_{R}+k_{P}\,\frac{{\left[C\,a^{2+}\right]}_{i}}{{\left[C\,a^{2+}\right]}_{i}+k_{\pi }}\right)}^{0.7}},$$

(21) $$J_{P\,L\,C\,\delta }=\left(v_{4}+k_{P\,L\,C\,\delta }\,a\right)\,\frac{{\text{[Ca}}^{2+}]{}_{\text{i}}+(1-\alpha )k{}_{4}}{{\text{[Ca}}^{2+}]{}_{\text{i}}+k{}_{4}},$$

where the parameters *k*_*PLCβ*_ and *k*_*PLCδ*_ control the strength of the linear influence of Aβ on each term, respectively.

All the parameters used in our model can be found in Table 2. In simulations, parameters are chosen under the principle that the oscillations obtained agree qualitatively with the experimental data. More details of the model can be found in the associated MATLAB code (R2020a, MathWorks; see in the Supporting Material).

**TABLE 2:** Parameters used in the model.

Parameter	Description	Value
*a*	A fixed level of Aβ concentration	≥ 0
*c_1_*	The ratio of ER volume to the cytoplasmic volume	0.185
*z*	Valence of Ca^2+^	2
*z_K_*	Valence of K^+^	1
*F*	Faraday constant	96,485 C/mole
*R*	Ideal gas constant	8.31 J/(moleK)
*T*	Temperature	293 K
*V*_ast_	The volume of an astrocyte	3.49*10^–13^ L
*v_1_*	Max Ca^2+^ channel flux	6 s^–1^
*v_2_*	Ca^2+^ leak flux constant	0.11 s^–1^
*v_3_*	Maximum SERCA pump flux	2.2 μM/s
*v_4_*	Maximum rate of IP_3_ production	0.5 μM/s
*v_5_*	Transmembrane leak flux	0.036 μM/s
*v*_β_	Maximal rate of IP_3_ production by PLCβ	0.05 μM/s
*k_0_*	Zero calcium concentration level leak from RyRs	0.013 s^–1^
*k_1_*	Rate constant of calcium extrusion	0.5 s^–1^
*k_2_*	Maximal rate of the RyRs	0.18 s^–1^
*k_3_*	Dissociation constant of Ca^2+^ to SERCA	0.05 μM
*k_4_*	Dissociation constant for Ca^2+^ stimulation of IP_3_ production	1.1 μM
*k_d_*	RyR sensitivity for the CICR	0.13 μM
*k_R_*	Glutamate affinity	1.3 μM
*k_P_*	The Ca^2+^/PLC-dependent inhibition factor	10 μM
*k*_π_	Ca^2+^ affinity of PLC	0.6 μM
*k*_*deg*_	Rate of IP_3_ degradation	1 s^–1^
*d_1_*	Dissociation constant for IP3	0.13 μM
*d_2_*	Inactivation dissociation constant of Ca^2+^	1.049 μM
*d_3_*	Inactivation dissociation constant of IP_3_	0.9434 μM
*d_5_*	Ca^2+^ activation constant	0.08234 μM
*a_2_*	Ca^2+^ inhibition constant	0.2 s^–1^
*k*	The cooperativity coefficient	4
*g*	Concentration of glutamate	1 μM
α	The relative effect of Ca^2+^ stimulation of PLCδ on IP_3_ production	0.8
*k*_*VGCC*_	The strength of the influence of Aβ on VGCCs	10
*k*_*in*_	The strength of the influence of Aβ on Aβ channels	1
*k*_*RyR*_	The strength of the influence of Aβ on RyRs	0.2
*k*_*PLCβ*_	The strength of the influence of Aβ on glutamate-dependent IP_3_ production	0.05
*k*_*PLCδ*_	The strength of the influence of Aβ on Ca^2+^-dependent IP_3_ production	0.5
[*K*^+^]_*o*_	Extracellular K^+^ concentration	3–5 mM
[*K*^+^]_*i*_	Intracellular K^+^ concentration	130 mM
ε	Modulation factor	17 mV
*g_T_*	Steady conductance of T-type channel	0.06 pS
*g_L_*	Steady conductance of L-type channel	3.5 pS
*g_N_*	Steady conductance of N-type channel	0.39 pS
*g_R_*	Steady conductance of R-type channel	0.2225 pS
[*Ca*^2 +^]_*o*_	Extracellular Ca^2+^ concentration	1.5 mM

### Sensitivity and Robustness Analysis

Robustness characterizes the ability to maintain performance in the face of perturbations while sensitivity characterizes the ability of living organisms to adequately react to certain stimulus. The two concepts are interlinked. In deterministic modeling, robustness is usually quantified by calculating sensitivity, e.g., period and amplitude sensitivity in quantifying robustness of circadian rhythms (Dubitzky et al., 2013). In this study, our model is highly sensitive to certain parameters and the oscillatory responses presented here only occur under certain scenarios. Therefore, a simple local sensitivity analysis was performed to assess the sensitivity of the model output, i.e., [Ca^2+^]_*i*_ and frequency, to variation in parameters determining the contribution of various fluxes and those representing the effect of therapy. We followed the Morris screening method in which only one input parameter *x_i_* is modified between two successive runs of the model. The change of the output induced onto the model objective function *y*(*x*)=(*x*_1_,*x*_2_,,*x*_*n*_), can then be unambiguously attributed to such a modification of *x_i_*. Therefore, the sensitivity *S* of an oscillatory amplitude and frequency to changes in model parameters *P* can be quantified by:


$$S=\frac{△\,O\,u\,t\,p\,u\,t}{△\,P_{c\,h\,a\,n\,g\,e}},$$

where △*Output* represents the changes of the model output induced by the changes in input parameters, and △*P*_*change*_ is the changes in input parameters between two runs of the model. Each parameter was allowed to vary around its control value and the model was solved for each parameter change.

## Results

Our simulations show that Aβ can trigger disruptions of cytosolic Ca^2+^ levels through interactions between various mechanisms, or, the single components. Each pathway exhibits different characteristics with the influence of Aβ. Changes in astrocyte resting membrane potential (RMP) were considered and incorporated into our model. With the intention of restoring dysregulated calcium signals, some therapeutic measures were tested, and the multi-pathway involved “combination therapy” gained effective recovery.

### Aβ Impairs Ca^2+^ Homeostasis in Astrocytes

**FIGURE 2:**
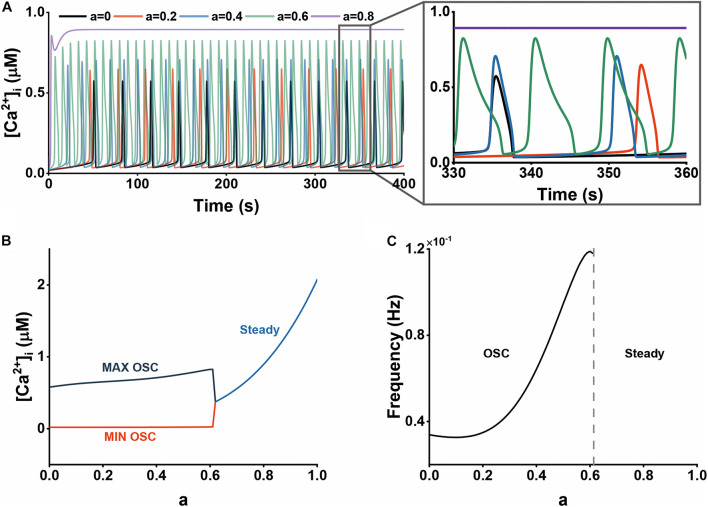
The effect of Aβ on cytosolic Ca^2+^ dynamics. **(A)** The time course of Ca^2+^ dynamics at different Aβ levels (0, 0.2, 0.4, 0.6, and 0.8). **(B)** Bifurcation diagram of [Ca^2+^]_*i*_ plotted against the Aβ level, *a*. **(C)** The frequency of Ca^2+^ oscillations plotted against the Aβ level, *a*.

Calcium oscillations in astrocytes are crucial signaling pathways with multiple roles in several brain functions (Riera et al., 2011a). Charles et al. (1991) have reported the [Ca^2+^]_*i*_ oscillations in glial cells with the peak amplitude of 0.6–0.8 μM induced by mechanical and glutamate stimulations. Parri and Crunelli (2001) found a subset of spontaneously active thalamic astrocytes exhibits [Ca^2+^]_*i*_ oscillations with an average frequency of 0.019 Hz. Here, our model produced typical calcium oscillations of approximately 0.6 μM in amplitude and 0.03 Hz in frequency without Aβ (Figure 2) which is consistent with the experimental data suggesting the physiological agreement of our biophysical model.

Aβ has been shown to impair intracellular Ca^2+^ homeostasis in our simulations. Figure 2A describes the cytosolic Ca^2+^ with different Aβ levels. We can clearly observe the increasing amplitude and frequency with elevated Aβ. The intracellular calcium signals would change into a high steady state if there is excessive Aβ. In Figures 2B,C, we performed analyses on bifurcation features and variations of frequency to study the dynamical influence of Aβ on Ca^2+^. At a low level of Aβ, that is, 0 < *a* < 0.3, which we assumed to represent an early or milder state of the disease, the oscillation amplitude increases while the frequency changes little. At a middle Aβ level (0.3 < *a* < 0.6), the oscillation frequency has a rapid increase. For a higher level of Aβ (*a* > 0.6), which represents a later or more severe stage of the disease, astrocytic Ca^2+^ dynamics become the high steady state in which the calcium concentration has an explosive increase.

**FIGURE 3:**
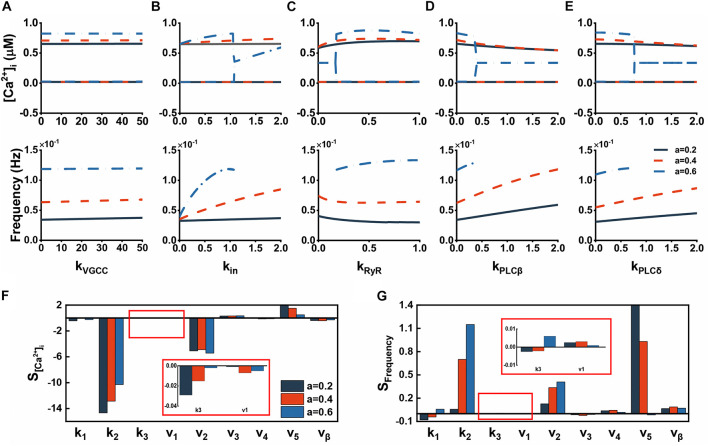
The effect of various parameters on cytosolic Ca^2+^ dynamics. **(A–E)** Bifurcation diagrams of [Ca^2+^]_*i*_ plotted against the strength of Aβ acting on different pathways. **(F)** Amplitude sensitivity of Ca^2+^ dynamics. The sensitivities are calculated for parameter variations of ± 10%. **(G)** Frequency sensitivity of Ca^2+^ dynamics. The sensitivities are calculated for parameter variations of ± 10%.

Since Aβ has such significant effects, we continued to test parameters representing the strength of Aβ on each pathway. Illustrated in Figures 3A–E are bifurcation analysis for this purpose. Regardless of the level of Aβ, the effect on VGCCs is minimal because both amplitude and frequency show good robustness against *k*_*VGCC*_ (Figure 3A). Nevertheless, Aβ channels makes an obvious influence with increasing Aβ. Both [Ca^2+^]_*i*_ and frequency markedly vary in oscillation or steady state (Figure 3B). Figure 3C displays the effect of *k*_*RyR*_, showing that [Ca^2+^]_*i*_ is relatively robust against large *k*_*RyR*_ whereas the frequency is quite the opposite. On balance, *k*_*RyR*_ induced alterations are minor. Figures 3D,E are the effects of parameters related to IP_3_ production and their influence is essentially similar. Increasing IP_3_ can make elevated frequency but slightly decreased [Ca^2+^]_*i*_. They both do not affect the steady state. Besides, we also considered other indirect parameters. Their sensitivities which are calculated for parameter variations of ± 10% are shown in Figures 3F,G. A small change in *k_2_* can cause a significant decrease in [Ca^2+^]_*i*_ and increase in frequency, while the rate of ER leakage (*v_2_*) acts as the same role but is less sensitive than *k_2_*. Another leak flux *J*_*in*_ has a positive effect on both sides. Some other parameters here with low sensitivity are not discussed further.

In general, various results suggest that Aβ triggers aberrant calcium signals by increasing [Ca^2+^]_*i*_ and frequency. In particular, high levels of Aβ are more hazardous and can be fatal. These disruptions of calcium oscillations make calcium signaling abnormal and raise the possibility of calcium intoxication (Mattson and Chan, 2003).

### Contribution of Aβ on Ca^2+^ Dynamics Through Different Pathways

The above results have revealed how the multiple Aβ-affected pathways interact and collectively contribute to Ca^2+^ dysregulation. However, we remained curious about the alterations in these pathways, and therefore, we next separated these pathways and examined, in isolation, how Aβ alters these pathways and causes abnormalities in cytosolic Ca^2+^. So, we denoted whether Aβ affects calcium flux by changing the strength of the effect of Aβ on the individual pathways (i.e., *k*_*VGCC*_, *k*_*in*_, *k*_*RyR*_, *k*_*PLCβ*_ and *k*_*PLCδ*_) to 0 or 1 and described the dynamics of varying the parameter *a*, meaning how Aβ acts on a single pathway.

**FIGURE 4:**
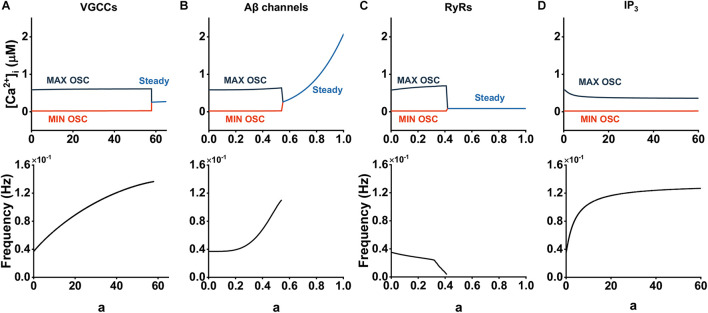
Contribution of Aβ on cytosolic Ca^2+^ dynamics through different pathways. **(A–D)** Bifurcation diagrams of [Ca^2+^]_*i*_ plotted against the Aβ level with affected pathways.

We examined the effect of each pathway on cytosolic Ca^2+^ dynamics by analyzing bifurcation and frequency. The results are shown in Figure 4. Diagram indicates that large amounts of Aβ are required to elicit changes in intracellular Ca^2+^ activity when acting on VGCCs; even at steady state, the effects of Aβ are slow (Figure 4A). This illustrates that [Ca^2+^]_*i*_ are robust to changes in *J*_*VGCC*_. In contrast, slight Aβ acting on the formed channels is able to disrupt normal astrocytic calcium oscillations and enables [Ca^2+^]_*i*_ at steady state to grow rapidly as well. When *a* < 0.3, the frequency essentially exhibits unchanged behavior. Then the frequency increased significantly as Aβ became larger. Figure 4C illustrates the effect of *J*_*RyR*_. We can observe the increased amplitude of oscillation, stable and unchanged low steady state concentration, and reduced frequency, especially when *a* > 0.3, where a sharp decrease in frequency occurs. Figure 4D shows that Aβ-mediated IP_3_ production leads to the decrease of calcium oscillation amplitude and the increase of frequency, but this effect gradually disappears with the increase of the Aβ level. We analyzed the reason and found that when Aβ increases to a certain value, both *m* and *n* tend to be constant, resulting in no change of *J*_*CICR*_ affected by IP_3_, so the whole dynamics gradually tend to be stable.

On the whole, we considered that Aβ channels and RyRs play a major role in the regulation of intracellular Ca^2+^ under the influence of Aβ, while the other two pathways have little effect.

### The Influence of Resting Membrane Potential

Although astrocytes are non-excitable cells, values of RMP reported in different studies may vary in extension to some ones due differences in outside K^+^ concentration (Anderson et al., 1995). Thus, the changes in extracellular K^+^ concentration can depolarize the astrocyte and increase the open probability of VGCCs (Bellot-Saez et al., 2017). Therefore, we simulated how changes in RMP can affect the *J*_*VGCC*_ and intracellular Ca^2+^ dynamics.

The hyperpolarized RMP of mature astrocytes is set close to the K^+^ Nernst potential, approximately –80 mV (Verkhratsky and Nedergaard, 2018). So, we modeled *V_m_* as the following form (Wu et al., 2019):


(22) $$V_{m}=\frac{R\,T}{z_{K}\,F}\,\ln\frac{{\left[K^{+}\right]}_{o}}{{\left[K^{+}\right]}_{i}}+\varepsilon ,$$

where *R* is the ideal gas constant, *T* is the absolute temperature, *z_K_* is the valence of K^+^, and *F* is Faraday constant. [K^+^]_*o*_ and [*K*]_*i*_ are the extracellular and intracellular K^+^ concentrations, respectively. ε is a modulation factor.

**FIGURE 5:**
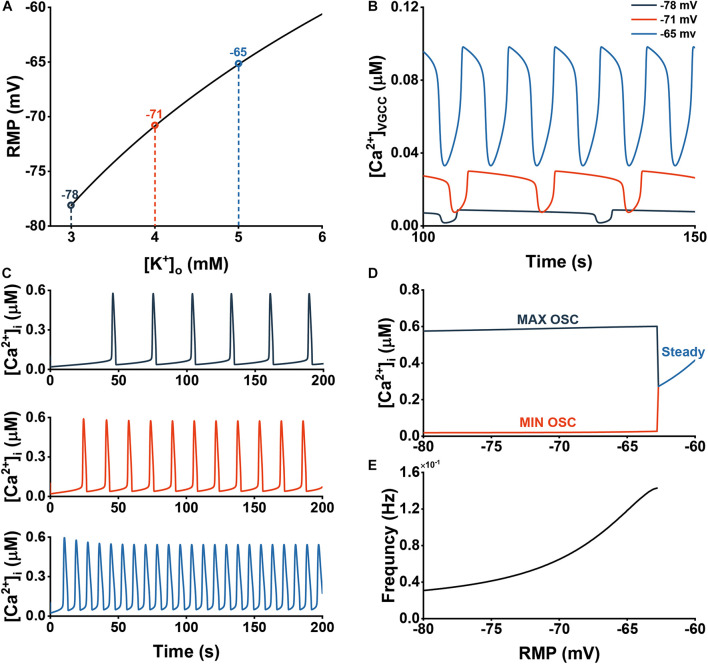
Extracellular K^+^ increases the frequency of Ca^2+^ oscillations through activation of VGCCs. **(A)** The dependency of the astrocytic RMP *V_m_* of different [K^+^]_*o*_. **(B)** Ca^2+^ influx through VGCCs at three different RMPs (–78, –71, and –65 mV). **(C)** Cytosolic Ca^2+^ dynamics at three different RMPs (–78, –71, and –65 mV). **(D)** Bifurcation diagram of the [Ca^2+^]_*i*_ in cytosol vs. RMP. **(E)** The frequency of the oscillations vs. RMP.

**FIGURE 6:**
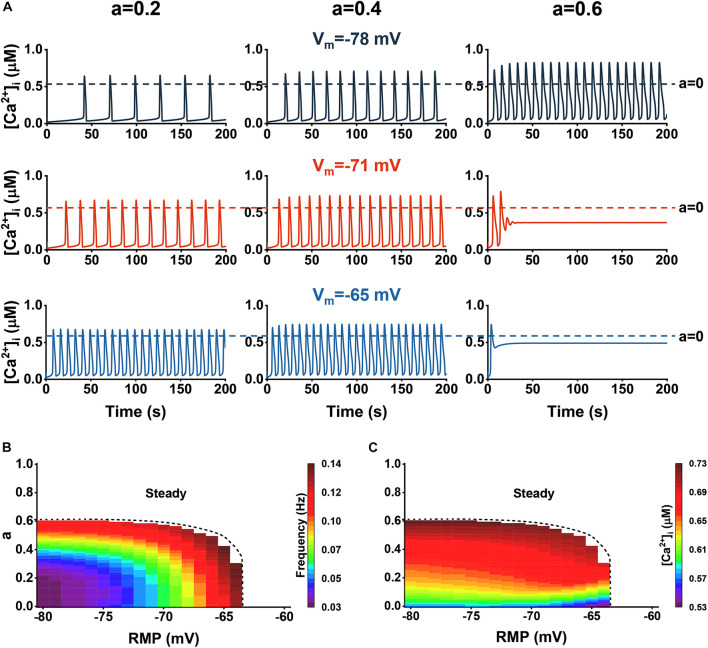
The influence of Aβ on Ca^2+^ dynamics with RMP. **(A)** Representative traces of Ca^2+^ dynamics within the three different Aβ levels (0.2, 0.4, and 0.6) at different *V_m_*: –78, –71, and –65 mV. **(B)** The frequency of Ca^2+^ signals vs. Aβ levels and RMPs. **(C)** The amplitude of Ca^2+^ signals vs. Aβ levels and RMPs.

Now we can examine the effects of changes to RMP by adjusting [*K*]_*o*_. The impact of such changes in *V_m_* and the resulting Ca^2+^ dynamics are shown in Figures 5, 6.

Figure 5A displays the dependence of *V_m_* on [*K*]_*o*_. We then chose three different values of *V_m_* with [*K*]_*o*_ of 3, 4, and 5 mM for our following simulations. Figure 5B shows how RMP significantly affect the *J*_*VGCC*_. Both the amplitude and frequency increase with depolarization. These alterations in *J*_*VGCC*_ similarly affected [Ca^2+^]_*i*_ (Figure 5C). Through the bifurcation and frequency diagrams in Figures 5D,E, we could observe that before reaching steady state, [Ca^2+^]_*i*_ is very robust to RMP, but after entering steady state, it increases dramatically. In contrast, the frequency consistently shows an increasing trend.

We induced Aβ and studied the interactions between the two factors. Figure 6A shows the cytosolic Ca^2+^ dynamics vs. three different RMP (−78, −71, and −65 mV) and three different Aβ levels (0.2, 0.4, and 0.6). In Figures 6B,C, we show the analysis of the frequency and amplitude of cytosolic Ca^2+^ dynamics, respectively. For the range where oscillation emerged (about −80 to −64 mV), the higher the RMP, the more profoundly affected by Aβ, since only a little Aβ is required to disrupt the oscillations. However, when at a high Aβ level, the range rapidly narrowed and eventually disappeared, indicating that the astrocytes will always be in the steady state with a high [Ca^2+^]_*i*_. In the research scope, increasing Aβ and RMP will lead to the increase of amplitude and frequency, in which the influence of the two on the amplitude is close while Aβ has a more significant contribution to the increase of calcium amplitude than the RMP.

### Blocking Aβ-Affected Pathways Benefits the Recovery of Calcium Homeostasis

**FIGURE 7:**
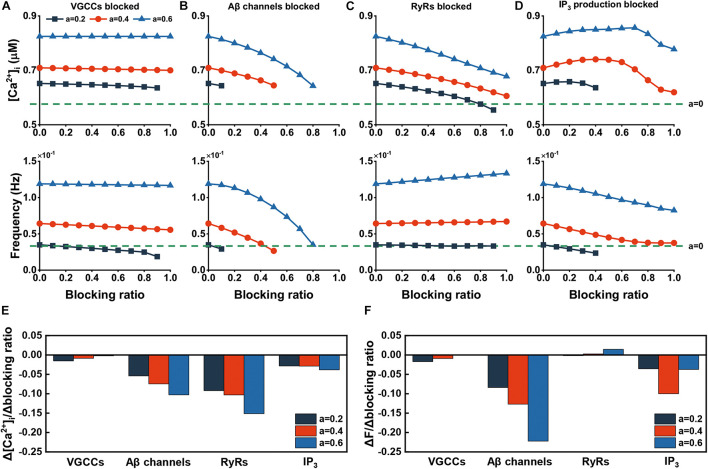
Effect of blocking channels, receptors, and products. **(A–D)** The changes in amplitude and frequency after different blocking ratios of related channels, receptors, and products. The green dashed line represents the absence of Aβ. **(E)** Amplitude sensitivity of recovery effect. The sensitivities are calculated by the slope in **(A–D)**. **(F)** Frequency sensitivity of recovery effect. The sensitivities are calculated by the slope in **(A–D)**.

At present, the treatment of AD remains a challenging research hotspot. There are four FDA-approved prescription drugs (Vaz and Silvestre, 2020) that show some effectiveness; however, they only relieve symptoms. Here, we tested the effects of blocking channels, receptors and products affected by Aβ as described above. We characterized the effect of recovery as a function of the blocking ratio of specific parameters and investigated their respective sensitivities at three different Aβ levels of *a* = 0.2, *a* = 0.4 and *a* = 0.6, which represented different stages of the disease. Results are shown in Figure 7.

If we blocked VGCCs, as you can see in Figure 7A, therapy of this pathway shows a weak effect to the recovery of [Ca^2+^]_*i.*_ For frequency, this method can restore the frequency to normal at the low level of Aβ (*a* = 0.2), but when at the high level, even the channel is completely blocked, it fails to do so. Inhibition of membrane leak flux obtains effective results with the ability to reduce the frequency and amplitude of abnormal rise (Figure 7B). Blocking RyRs has a certain effect on the recovery of elevated [Ca^2+^]_*i*_, but the recovery is not obvious and even has an adverse effect at a higher Aβ level for frequency (Figure 7C). Reducing the production rate of IP_3_ can help to low down the increased frequency. However, this way contributes to decreasing [Ca^2+^]_*i*_ only at high inhibition ratio (Figure 7D). Illustrated in Figures 7E,F are quantitative sensitivity analysis of the recovery of [Ca^2+^]_*i*_ and frequency, respectively. Overall, targeting *J*_*in*_ has the best effect, followed by RyR blockade, and blocking VGCCs has the least effect.

**FIGURE 8:**
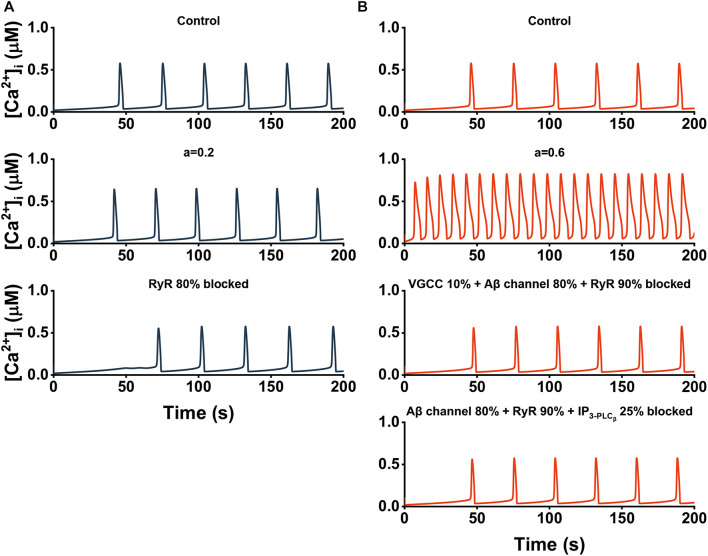
The cytosolic Ca^2+^ dynamics after treatment. **(A)** Effect of single therapy at the early stage of AD. Top, *a* = 0. Middle, *a* = 0.2. Bottom, *a* = 0.2 + single therapy. **(B)** Effect of “combination therapy” at the advanced stage of AD. Top, *a* = 0. Middle, *a* = 0.6. Bottom, *a* = 0.6 + two different “combination therapies.”

In Figure 8, different strategies based on our sensitivity analysis are applied to restore calcium homeostasis for two different situations (*a* = 0.2 and *a* = 0.6). When *a* = 0.2 representing the early stage of the disease, there exists significant variation in frequency. However, the abnormal signals can recover to some extent by simply inhibited the RyR pathway by 80% (Figure 8A). When *a* = 0.6 representing the advanced stage of the disease in which both frequency and [Ca^2+^]_*i*_ are markedly affected, targeting only one pathway is powerless. With the “combination therapy”, we can not only get the ideal recovery effect, but also carry out the treatment with more than one strategy (Figure 8B).

## Discussion

With a rapidly aging population, AD has become a major public health concern. However, the causation of AD remains unclear. Dysregulation of astrocytic Ca^2+^ has been widely regarded as an important component of AD. While controversy still remains, because some researches have not found acute [Ca^2+^]_*i*_ responses to Aβ (Casley et al., 2009; Toivari et al., 2011), this could possibly be attributed to the variability of Aβ species (monomers, oligomers, fibrils) and astrocyte types (from different brain areas) (Lim et al., 2014). More experiments reported that Aβ triggered transient [Ca^2+^]_*i*_ increases or [Ca^2+^]_*i*_ oscillations in astrocytes (Jalonen et al., 1997; Abramov et al., 2003; Alberdi et al., 2013; Lim et al., 2014). Unfortunately, the interactions of various mechanisms make it difficult to precisely understand how Aβ impacts cytosolic Ca^2+^ levels and individual fluxes. Decoupling various components and integrating their contributions into a full view may help us better understand the complexity of Aβ mechanisms in intracellular calcium dysregulation.

Computational modeling is a powerful approach that provides great opportunities to study complex mechanisms since it is experimentally difficult to isolate each component for separate studies. Our previous work on bioRxiv (Gao et al., 2020) has proposed a model related to AD focusing on the effect of Aβ through four different pathways on cytosolic Ca^2+^. Although we have considered a multi-pathway model, there is still room for more in-depth study of the individual contribution of Aβ on each pathway and methods of restoring the disrupted signals. There are also some limitations to the model itself. Specifically, for example, our prior study has only included the PLCδ-dependent production while IP_3_ is regulated by two entirely different pathways in astrocytes. Also, differences in volume of cytosol and ER, and changes in membrane potential were neglected. These all deserve rigorous consideration and continued refinement.

In this paper, we modified and extended the previous studies in conjunction with experimental data and a comprehensive model was proposed. Not only all the issues mentioned are considered, but also the model is more physiologically reasonable. Just as important, the brevity and universality of the modeling method are worth noting. We previously used Lavrentovich’s model (Lavrentovich and Hemkin, 2008) to describe CICR dynamics depended on [IP_3_] and [Ca^2+^]_*i*_ from both the ER and cytosol. However, a form simplified from De Young’s model (De Young and Keizer, 1992) by Li (Li and Rinzel, 1994) based on the gating of IP_3_R provides good agreement with experimental recordings of channel opening and is more suitable for biophysical modeling. These, then, make a model more compatible with physiological data, providing good foundations for simulation studies of AD-induced dysregulation of astrocytic Ca^2+^.

In the experimental studies, Jalonen et al. (1997) exposed astrocytes to 1 μM Aβ and found that transient increase in [Ca^2+^]_*i*_ rose from about 0.6 to 0.9 μM. Takano et al. (2007) reported that after the injection of Aβ in both Dutch/Iowa mice and controls, the frequency of astrocytic Ca^2+^ oscillations increased significantly and became approximately three times as the control group. In our model, similar results were observed. The [Ca^2+^]_*i*_ and frequency are about 0.83 μM and 0.12 Hz when *a* = 0.6, compared to 0.58 μM and 0.034 Hz when *a* = 0. This means that our model has a good physiological agreement. Besides, we also found that the accumulation of Aβ not only leads to the increase of [Ca^2+^]_*i*_ and frequency in astrocytes, but also makes a transition from oscillation to a high steady state after exceeding a certain threshold, in which, the intracellular [Ca^2+^]_*i*_ will remain at a very high level, and this level will continue to rise rapidly with the increase of Aβ. In AD, the high intracellular [Ca^2+^]_*i*_ directly impacts memory formation and consolidation (Berridge, 2014).

Upregulation of astrocytic VGCCs expression in astrocytes has been indicated in pathological conditions, especially the L-Type in AD. Our model shows that VGCCs may contribute less to the dysregulation of cytosolic Ca^2+^ levels. This is because [Ca^2+^]_*i*_ is very robust either under high strength of the effect of Aβ or a large amount of Aβ. Even when there is a clear change in frequency, the sensitivity is low relative to the changes in Aβ. In response to the disruptions, Anekonda et al. (2011) have demonstrated that L-type Ca^2+^ current blockers can protect cells from the inductive effect of Aβ. We did confirm the efficacy of blocking VGCCs, but the recovery was not significant enough.

Aβ has been shown to impair the membrane permeability, due to the Aβ-formed pores resulting in the increase of Ca^2+^ influx through the membrane (Demuro et al., 2010). This additional influx shows damage to astrocytic homeostasis in our simulations. Especially, when oscillations disappear and the astrocyte calcium level is at a high steady state, Aβ can cause dramatic increases in [Ca^2+^]_*i*_. In fact, channel formation has been already proposed as a molecular mechanism for Aβ toxicity in the early 1990s (Arispe et al., 1993), and our model, in the light of physiology, has well reflected how ionic leakage strongly affects and rapidly disrupts the cellular homeostasis. This intense pathology can be treated with Zn^2+^ (Abramov et al., 2003). We demonstrated that blocking the channel achieves desirable effects and may serve as a promising therapeutic approach.

Although disruptions on the membrane by Aβ are believed to be an important mechanism, the intracellular signaling pathways also deserve attention. In this study, we reflect the toxic effects of Aβ by increasing the channel open probability of RyRs. Other studies also suggested that Aβ can directly increase the RyR expression (Supnet et al., 2006). The increasing [Ca^2+^]_*i*_ and unchanged low steady state were observed. Unlike the other pathways, Aβ caused a decrease in frequency, which was more rapid in the presence of large amounts of Aβ because of the occurrence of mixed oscillations. Research has reported that RyR-mediated Ca^2+^ release can be reduced after treatment with RyR inhibitor (Oulès et al., 2012). Results showed a significant therapeutic effect on abnormal high [Ca^2+^]_*i*_ in our study.

Accumulated evidence indicates that Aβ is involved in the regulation of IP_3_ production in AD (Demuro and Parker, 2013; Jensen et al., 2013). In this paper, we considered both Aβ-mediated channels or receptors, as well as non-directly acting intermediates such as IP_3_, which embodies the concept of “multi-pathway” in a true sense and illustrates the complexity of our model. According to the results of the simulation, increasing IP_3_ levels can lead to the decrease of [Ca^2+^]_*i*_ but the increase of frequency. The recovery effect is stronger than the blockage of VGCCs but not as effective as the other two pathways. Reducing the intracellular IP_3_ level has a certain effect on frequency recovery, but the recovery effect on [Ca^2+^]_*i*_ is not ideal. This may reveal that IP_3_ mainly regulates the frequency of intracellular calcium signals.

The RMP of astrocytes is also a contributing factor within our consideration. Compared to their neuronal counterparts, astrocytes display a highly negative RMP (Bolton et al., 2006). Some studies have shown that [K^+^]_*o*_ is critical in establishing the RMP of astrocytes (Anderson et al., 1995; Bellot-Saez et al., 2017). However, defective extracellular K^+^ clearance mechanisms have also been observed in AD which led to the loss of astrocyte polarization (Olabarria et al., 2010). Our model shows that increasing [K^+^]_*o*_ contributes to astrocyte depolarization, which to some extent reflects the impairment of astrocyte polarization by abnormal accumulation of [K^+^]_*o*_ in AD. During the process of depolarization, the amplitude of transmembrane Ca^2+^ flow mediated by VGCCs, as well as frequency increases. The affected *J*_*VGCC*_, in turn, will cause further damage to cytosolic Ca^2+^ dynamics, manifested primarily by a large increase in [Ca^2+^]_*i*_ at steady state and a marked increase in frequency. This finding suggests that the frequency of Ca^2+^ events can be increased by depolarization of astrocytes, through activating of VGCCs. On the other hand, the accumulation of Aβ can narrow the range of membrane potential where oscillations are triggered. This may indicate the destructive nature of Aβ to synchronization oscillation of astrocyte network. Besides, astrocyte RMP displays extensive heterogeneity in the central nervous system (McNeill et al., 2021). To a certain extent, our model also reflects the characteristics of astrocytes with different RMP.

Finding a cure for AD is one of the most urgent and difficult tasks in modern medicine. At present, the drugs that are used to treat AD can only relieve symptoms but cannot slow down or reverse the progression of the disease (Alzheimer’s Association, 2020). The recent development and FDA approval of the AD drug Aducanumab, which targets Aβ, caused a great controversy (Vaz and Silvestre, 2020). But there is no doubt that drug research targeting Aβ or Tau proteins has once fallen into a bottleneck (Congdon and Sigurdsson, 2018; Foroutan et al., 2019), so it is advisable to explore feasible therapies from the perspective of Ca^2+^ dynamics. Mounting evidence has demonstrated that calcium signals play an indispensable role in AD (Yu et al., 2009), but Ca^2+^-pathway therapeutics remain undeveloped. Our simulations suggested that therapy targeting a specific receptor, channel or product is efficacious but limited because of multiple targets of Aβ, particularly in the terminal stages of the disease. Meanwhile, combination therapy can perfectly compensate for the shortage of single therapy. Generally, restoring calcium homeostasis is useful and necessary (Lim et al., 2014). Memantine, approved for the treatment of AD, has been clinically proven to be effective in preventing NMDAR-mediated calcium flux for decades, and here, we have demonstrated that other Ca^2+^ pathways in astrocytes may also be potential therapeutic targets and unraveled which Ca^2+^ pathway is effective because it is also likely to bring significant side-effects if the pathway is not carefully chosen.

It is challenging to establish a model of Aβ-mediated multi-pathway calcium dynamics. Although several mechanisms by which Aβ affects astrocytes have been experimentally demonstrated, how some of these mechanisms occur remains unclear. But the ubiquitous Ca^2+^ regulatory fluxes used in our model make it easily applicable for studying various cell types with spatial components. In fact, astrocytes exhibit a very complex morphology suggesting the spatiotemporal characteristics of Ca^2+^ signals in different structures (Semyanov et al., 2020) while we considered a spatially homogeneous astrocyte for better quantifying. However, even by adopting some simplifications, our model includes a large number of parameters, some of which still lack the effectiveness of the verification experiment. Our deterministic model generated regular calcium oscillations, which is consistent with the situation in some reports (Parri and Crunelli, 2001; Tashiro et al., 2002), However, in most cases, the calcium signals observed in the experiments presented clear irregularities which revealed its stochastic nature, meaning that some level of stochasticity may be closer to the physiology. All the deficiencies can be altered in future follow-up work as we continue to improve our understanding of the effects of Aβ in the astrocytic system. Our codes are publicly available for reproducibility, assisting with fast, convenient, and accurate validation of the model. Moreover, continuous improvement of the model combined with experimental data helps to make the model more useful.

In general, we have presented a hypothetical AD-specific model regarding Ca^2+^ dysregulation in astrocytes. The proposed general model incorporates multiple critical individually modeled Ca^2+^ mechanisms into a single framework, which obtains a more comprehensive picture compared to present work, especially fewer models are containing VGCCs and RyRs in astrocytes. To our knowledge, this is one of the few computational models to investigate the contribution of various Ca^2+^ fluxes to Ca^2+^ dynamics including entry and release under the influence of Aβ. Furthermore, we tested methods of blocking affected pathways. The “combination therapy” was first proposed and showed the significant effects on restoring calcium homeostasis. This may provide factual predictions for future drug development. Our study can provide an in-depth understanding of AD and pave the way toward the development of much more effective treatment modalities.

## Data Availability Statement

The original contributions presented in the study are included in the article/Supplementary Material, further inquiries can be directed to the corresponding author/s.

## Author Contributions

SC, HG, and AZ conceived and designed the research. LL and HG conducted literature research and wrote MATLAB code. LL, SC, and HG performed simulations and analyzed data and discussed the results and wrote the article with input from AZ. SC supervised the study. LL plotted pictures. All authors contributed to the article and approved the submitted version.

## Conflict of Interest

The authors declare that the research was conducted in the absence of any commercial or financial relationships that could be construed as a potential conflict of interest.

## Publisher’s Note

All claims expressed in this article are solely those of the authors and do not necessarily represent those of their affiliated organizations, or those of the publisher, the editors and the reviewers. Any product that may be evaluated in this article, or claim that may be made by its manufacturer, is not guaranteed or endorsed by the publisher.
