# Supplementary material for: Unraveling Aβ-Mediated Multi-Pathway Calcium Dynamics in Astrocytes: Implications for Alzheimer’s Disease Treatment From Simulations
Source: Front Physiol. 2021 Oct 28;12:767892. doi: 10.3389/fphys.2021.767892 (PMC8581622; doi:10.3389/fphys.2021.767892)
Supplement: Supplementary file 1 [file Data_Sheet_1.docx]

%% Unraveling the Abeta-mediated multi-pathway calcium dynamics in astrocytes: Implications for AD treatment for simulations

%% Authors: Shangbin Chen, Huayi Gao and Langzhou Liu

%% Please contact us for any questions.

%% Email: liulangzhou@hust.edu.cn

%% Set simulation conditions

tic; % Record the computing time

T=200; % Total time of simulation

dt=0.01; % Time step interva

ts=0:dt:T; % Time series

%% Parameters

% The parameters of this part are constants, which will not change in the whole simulation process.

% These parameters can also be adjusted according to your own needs

kVGCC=10; % The strength of the influence of Abeta on VGCCs

kin=1; % The strength of the influence of Abeta on abeta channels

kRyR=0.2; % The strength of the influence of Abeta on RyRs

kPLCb=0.05; % The strength of the influence of Abeta on glutamate-dependent IP3 production

kPLCd=0.5; % The strength of the influence of Abeta on Ca2+-dependent IP3 production

F=96485; % Faraday's constant, unit: coul/mole

R=8.31; % Gas constant, unit: J/(mole*K)

z=2; % Valence of Ca2+

Tp=293; % Temperature

Vast=3.49*1e-13; % Volume of an astrocyte, unit: liter

c1=0.185; % The ratio of ER volume to the cytoplasmic volume

Cout=1500; % Ca2+ concentration in ECS, unit: uM

Kin=130; % K+ concentration in ICS, unit: mM

Kout=3; % K+ concentration in ECS, unit: mM

g=1; % External glutamate concentration, unit: uM

gT=0.0600; % Steady conductance of T type channel, unit: pS

gL=3.5000; % Steady conductance of L type channel, unit: pS

gN=0.3900; % Steady conductance of N type channel, unit: pS

gR=0.2225; % Steady conductance of R type channel, unit: pS

v1=6; % Max Ca2+ channel flux unit: s-1

v2=0.11; % Ca2+ leak flux constant unit: s-1

v3=2.2; % Maximum SERCA pump flux unit: uM/s

v4=0.5; % Maximum rate of IP3 production unit: uM/s

v5=0.036; % Transmembrane leak flux uM/s

vb=0.05; % Maximal rate of IP3 production by PLC¦Â unit: uM/s

k0=0.013; % Zero calcium concentration level leak from RyRs unit: s-1

k1=0.5; % Rate constant of calcium extrusion unit: s-1

k2=0.18; % Maximal rate of the RyRs unit: s-1

k3=0.05; % Dissociation constant of Ca2+ to SERCA unit: uM

k4=1.1; % Dissociation constant for Ca2+ stimulation of IP3 production unit: uM

kd=0.13; % RyR sensitivity for the CICR unit: uM

kp=10;% The Ca2+/PLC-dependent inhibition factor unit: uM

kR=1.3; % Glutamate affinity unit: uM

kpi=0.6; % Ca2+ affinity of PLC unit: uM

kdeg=1; % Rate of IP3 degradation unit: s-1

d1=0.13; % Dissociation constant for IP3 unit: uM

d2=1.049; % Inactivation dissociation constant of Ca2+ unit: uM

d3=0.9434; % Inactivation dissociation constant of IP3 unit: uM

d5=0.08234; % Ca2+ activation constant unit: uM

a2=0.2; % Ca2+ inhibition constant unit: s-1

m=4; % Cooperativity coefficient

alpha=0.8;

delta=17; % Modulation factor unit: mV

%% VGCCs Gating

V=R*Tp/F*1e3*log(Kout/Kin)+delta;

ml=1/(1+exp(-(V+50)/3));

mn=1/(1+exp(-(V+45)/7));

mr=1/(1+exp(-(V+10)/10));

hr=1/(1+exp((V+48)/5));

mt=1/(1+exp(-(V+63.5)/1.5));

htf=1/(1+exp((V+76.2)/3));

hts=1/(1+exp((V+76.2)/3));

%% Initial

% This part is to initialize the matrix and variables

freqs=[]; % Frequency series

mps=[]; % Abeta levels series

count=0; % For counting

x=0; % For counting

Cin(1)=0.1; % Initial Ca2+ concentration in cytosol

Cer(1)=1.5; % Initial Ca2+ concentration in ER

IP3(1)=0.1; % Initial IP3 concentration

h(1)=0.78;

%% Simulation

for aa=0:1:100

a=aa/100;

for t=1:length(ts)-1

alphah=a2*d2*(IP3(t)+d1)/(IP3(t)+d3);

betah=a2*Cin(t);

minf=IP3(t)/(IP3(t)+d1);

ninf=Cin(t)/(Cin(t)+d5);

hl(t)=0.00045/(0.00045+Cin(t)/1000);

hn(t)=0.0001/(0.0001+Cin(t)/1000);

Eca(t)=R*Tp/2/F*1e3*log(Cout/Cin(t));

IL(t)=(gL+kVGCC*a)*ml*hl(t)*(V-Eca(t));

IN(t)=gN*mn*hn(t)*(V-Eca(t));

IR(t)=gR*mr*hr*(V-Eca(t));

IT(t)=gT*mt*(htf+0.04*hts)*(V-Eca(t));

IVGCC(t)=(IN(t)+IL(t)+IR(t)+IT(t));

JPLCb(t)=(vb+kPLCb*a)*g^0.7/(g^0.7+(kR+kp*Cin(t)/(Cin(t)+kpi))^0.7);

JPLCd(t)=(v4+kPLCd*a)*(Cin(t)+(1-alpha)*k4)/(Cin(t)+k4);

JVGCC(t)=(-IVGCC(t)*1e4/(3.49)/(2*F));

JRyR(t)=(k0+((k2*Cin(t)^3)/((kd+kRyR*a)^3+Cin(t)^3)))*(Cer(t)-Cin(t));

Jpm(t)=k1*Cin(t);

Jin(t)=v5+kin*a^m;

JCICR(t)=v1*minf^3*ninf^3*h(t)^3*(Cer(t)-Cin(t));

Jleak(t)=v2*(Cer(t)-Cin(t));

JSERCA(t)=v3*Cin(t)^2/(Cin(t)^2+k3^2);

Cin(t+1)=dt*((JVGCC(t)-Jpm(t)+Jin(t))+JCICR(t)-JSERCA(t)+JRyR(t)+Jleak(t))+Cin(t);

Cer(t+1)=dt*((JSERCA(t)-JCICR(t)-JRyR(t)-Jleak(t))/c1)+Cer(t);

IP3(t+1)=dt*((JPLCb(t)+JPLCd(t))-kdeg*IP3(t))+IP3(t);

h(t+1)=dt*(alphah*(1-h(t))-betah*h(t))+h(t);

end

% Plot Ca oscillations in cytosol at different Abeta levels

if(mod(a,0.2)==0 && a<=0.6)

set(gcf,'unit','normalized','position',[0.25 0.25 0.5 0.5])

x=x+1;

subplot(2,2,x);plot(Cin,'k','LineWidth',2)

set(gca,'xLim',[0,T/dt],'yLim',[0,1],'FontSize',20,'LineWidth',2,'Fontweight','bold')

xticks(linspace(0,length(Cin)-1,3))

xticklabels(linspace(0,length(Cin)-1,3)/100)

title(['a=',num2str(a)],'FontSize',20,'Fontweight','bold')

ylabel('[Ca^{2+}]','FontSize',20)

xlabel('Time (s)','FontSize',20)

pause(0.1)

hold on

end

% Bifurcation

warning off; % Cancel the warning of no wave crest in non oscillation state

[maxv,maxl]=findpeaks(Cin,'minpeakheight',0.4); % Find the abscissa and ordinate corresponding to the first and second wave crest

lmaxv=length(maxv);

dele=[];

for j=1:lmaxv

if (Cin(maxl(j))-Cin(maxl(j)-100))<0.0001

dele=[dele,j];

end

end

maxv(dele)=[];maxl(dele)=[];

if length(maxv)>=5 % In order to ensure that the oscillation is stable

Cmin(aa+1)=min(Cin);

Cmax(aa+1)=max(Cin);

end

if length(maxv)<5

Cmin(aa+1)=Cin(t);

Cmax(aa+1)=Cin(t);

end

% % Calculate the frequency

if length(maxv)>=5

count=count+1;

mps(count)=aa;

freq=1/((maxl(2)-maxl(1))/100);

freqs=[freqs freq];

end

end

toc % Show the computing time

% Plot bifurcation diagram

figure;

set(gcf,'unit','normalized','position',[0.25 0.25 0.5 0.5])

plot(1:81,Cmin(1:81),'k','LineWidth',2);

hold on;

plot(Cmax,'k','LineWidth',2);

set(gca,'XLim',[1,81],'YLim',[0,1],'LineWidth',2,'Fontweight','bold','FontSize',20);

xticks(linspace(0,80,9))

xticklabels(linspace(0,0.8,9))

ylabel('[Ca^{2+}]')

xlabel('Abeta level')

% Plot the relationship between frequency and Abeta level

mpx=mps(1):1:mps(count);

figure;

set(gcf,'unit','normalized','position',[0.25 0.25 0.5 0.5])

values = spcrv([[mpx(1) mpx mpx(end)];[freqs(1) freqs freqs(end)]],3);

plot(values(1,:),values(2,:),'k','LineWidth',2);

set(gca,'LineWidth',2,'Fontweight','bold','FontSize',20);

xticks(linspace(0,100,11))

xticklabels(linspace(0,1,11))

title('F-a')

ylabel('Frequency (Hz)')

xlabel('Abeta level')
